# Hyaluronic Acid in Liver Fibrosis: Role in Inflammation, Tissue Remodeling, and Disease Progression

**DOI:** 10.3390/ijms262010139

**Published:** 2025-10-18

**Authors:** Carlos Rojano-Alfonso, Cristina López-Vicario, Berta Romero-Grimaldo, Bryan J. Contreras, Joan Clària, Esther Titos

**Affiliations:** 1 Biochemistry and Molecular Genetics Service, Hospital Clínic de Barcelona, Fundació de Recerca Clínic Barcelona–Institut d’Investigacions Biomèdiques August Pi i Sunyer (IDIBAPS), 08036 Barcelona, Spain; rojano@recerca.clinic.cat (C.R.-A.); clopezv@recerca.clinic.cat (C.L.-V.); romerog@recerca.clinic.cat (B.R.-G.); bjcontreras@recerca.clinic.cat (B.J.C.); jclaria@clinic.cat (J.C.); 2 European Foundation for the Study of Chronic Liver Failure (EF CLIF) and Grifols Chair, 08021 Barcelona, Spain; 3 Centro de Investigación Biomédica en Red de Enfermedades Hepáticas y Digestivas (CIBERehd), 28029 Madrid, Spain; 4 Department of Biomedical Sciences, University of Barcelona, 08036 Barcelona, Spain; 5 Molecular Biology CORE, Hospital Clínic de Barcelona, 08036 Barcelona, Spain

**Keywords:** liver fibrosis, MASLD, hyaluronic acid, extracellular matrix, inflammation, DAMPs

## Abstract

Hyaluronic acid (HA) is a major glycosaminoglycan in the hepatic extracellular matrix and pericellular space, playing a critical role in maintaining liver architecture and regulating cell–matrix interactions. In chronic liver disease, regardless of etiology, dysregulated HA metabolism, particularly the generation and accumulation of low-molecular-weight HA (LMW-HA), has been implicated in fibrogenesis, immune dysregulation, and hepatocellular carcinogenesis via receptor-mediated pathways involving lymphocyte homing receptor (CD44), receptor for hyaluronan-mediated motility (RHAMM), and Toll-like receptors (TLRs). This review synthesizes current evidence on HA biosynthesis, turnover, and signaling, emphasizing its dual role as a structural scaffold and as an active modulator of immune responses and tumor progression in chronic liver disease. Given the rising global burden of metabolic liver disease, and in line with our recent findings that small HA fragments are elevated in obesity and promote low-grade, TLR-dependent activation of innate immune cells, we emphasize metabolic dysfunction-associated steatotic liver disease (MASLD) as a highly prevalent and clinically relevant setting to examine HA-driven immunomodulation during progression to advanced fibrosis and hepatocellular carcinoma (HCC) and to consider therapeutic strategies targeting HA synthesis, turnover, or receptor signaling.

## 1. Introduction

Hyaluronic acid (HA) is a naturally occurring glycosaminoglycan widely distributed throughout human tissues, serving as a key structural and functional component of the extracellular matrix (ECM). HA also contributes to the glycocalyx, the thin, carbohydrate-rich pericellular layer present on the surface of cells [1]. In the healthy liver, HA plays a crucial role in preserving parenchymal architecture, maintaining tissue hydration, and regulating immune tolerance and homeostasis [2,3]. However, in response to pathological stimuli such as viral hepatitis, alcohol abuse, metabolic disorders, or cholestatic and autoimmune conditions, HA metabolism becomes dysregulated. Under these conditions, HA transitions from being a mere structural regulator to becoming an active driver of hepatic inflammation, fibrogenesis, and carcinogenesis [4]. Consequently, elevated levels of serum HA are commonly detected in patients with chronic liver disease and are widely recognized as a reliable non-invasive biomarker for assessing liver fibrosis and cirrhosis. Indeed, serum HA levels have been integrated into several validated fibrosis scoring algorithms, such as the Enhanced Liver Fibrosis (ELF) test, FibroTest, and Hepascore, highlighting their clinical utility in staging disease severity and predicting prognosis [5,6]. This review aims to provide a comprehensive overview of the immunomodulatory functions of HA in liver pathophysiology, with a particular emphasis on its role in driving fibrosis progression and its emerging relevance in highly prevalent liver disease conditions, including metabolic dysfunction-associated steatotic liver disease (MASLD), steatohepatitis (MASH), and hepatocellular carcinoma (HCC).

## 2. The Molecular Size of HA Dictates Distinct Biological Functions

Over recent decades, comprehensive studies on HA metabolism and function have significantly expanded our understanding of its diverse biological roles, extending far beyond its traditional association with hydration and lubrication within the ECM. It is now well established that HA exists in tissues and biological fluids as a polydisperse population of polymers with a broad range of molecular weights. These variants are compartmentalized into distinct functional pools, including circulating HA in plasma, matrix-associated HA within the ECM, and pericellular HA contributing to the glycocalyx structure. Each pool plays context-specific roles in tissue hydration, structural integrity, cell signaling, and immune modulation, particularly relevant in liver physiology and pathology. Notably, in vitro studies using size-defined HA fractions have demonstrated that molecular weight critically influences HA’s ability to modulate cell behavior, immune signaling, and ECM dynamics, especially in inflammatory and fibrotic liver diseases [7,8,9]. The signaling pathways triggered by HA depend critically on its molecular weight, with high-molecular-weight HA (HMW-HA, >1000 kDa) and low-molecular-weight HA (LMW-HA, <500 kDa) often engaging the same receptors but eliciting divergent cellular responses [10].

HA molecules exert their biological effects through several receptors and binding proteins. These include well-characterized HA-specific receptors such as CD44 (also known as the lymphocyte homing receptor), receptor for hyaluronan-mediated motility (RHAMM, gene name *HMMR*), and lymphatic vessel endothelial hyaluronan receptor 1 (LYVE-1), as well as non-specific HA receptors such as Toll-like receptors (TLRs), particularly TLR2 and TLR4 [2]. Among these, CD44, RHAMM, and TLRs are considered the most functionally significant in liver physiology and pathology (Table 1).

CD44, cluster of differentiation 44; DAMP, damage-associated molecular pattern; ECM, extracellular matrix; HA, hyaluronic acid; HARE/Stabilin-2, hyaluronan receptor for endocytosis/Stabilin-2; HSCs, hepatic stellate cells; ICAM-1, intercellular adhesion molecule-1; LMW, low molecular weight; LSECs, liver sinusoidal endothelial cells; LYVE-1, lymphatic vessel endothelial hyaluronan receptor 1; MDSC, myeloid-derived suppressor cells; NF-κB, nuclear factor kappa B; RHAMM, receptor for hyaluronan-mediated motility; SHAPs, serum-derived hyaluronan-associated proteins; TLR2, Toll-like receptor 2; TLR4, Toll-like receptor 4.

CD44, a broadly expressed transmembrane glycoprotein, mediates HA-dependent cell adhesion, migration, and signal transduction, functions especially critical in hepatic stellate cells (HSCs) and immune cells. Beyond its interaction with HA, CD44 also binds other ECM components, including osteopontin, type I collagen, fibronectin, and matrix metalloproteinases (MMPs), as well as forming complexes with co-receptors such as integrins and receptor tyrosine kinases. This multifunctional binding landscape positions HA-CD44 as a critical regulatory axis in ECM remodeling and cellular crosstalk during liver injury and repair [19]. RHAMM is present at low or undetectable levels under physiological conditions, whereas its expression increases during tissue injury and inflammation, particularly in endothelial cells, smooth muscle cells, and macrophages [20,21,22]. Both receptors participate in physiological tissue repair and are implicated in pathological processes, including chronic liver inflammation and HCC. Mechanistically, CD44 and RHAMM activate intracellular signaling pathways such as mitogen-activated protein kinase (MAPK)/extracellular signal-regulated kinase (ERK) and phosphatidylinositol *3*-kinase (PI3K)/protein kinase B (AKT), which regulate cell proliferation, survival, and differentiation [4].

TLR2 and TLR4, in turn, function as promiscuous pattern recognition receptors and play crucial roles in liver immune surveillance. These receptors are primarily expressed in the liver by resident hepatic immune cells such as Kupffer cells (KCs) and other leukocytes. Still, they are also found in HSCs, liver sinusoidal endothelial cells (LSECs), and, to a lesser extent, hepatocytes. Both receptors signal via the myeloid differentiation primary response 88 (MyD88)-dependent pathway, leading to nuclear factor kappa B (NF-κB) activation and transcription of pro-inflammatory cytokines. Additionally, TLR4 signals through the TIR-domain-containing adapter-inducing interferon beta (TRIF)-dependent pathway in the endosomal compartment, inducing type I interferon responses and further amplifying hepatic inflammation [15].

Under physiological conditions, HMW-HA is the predominant form and is generally associated with anti-inflammatory and protective functions. HMW-HA maintains homeostasis through interactions with canonical receptors such as CD44 and RHAMM, supporting tissue integrity and immune quiescence [23,24]. In contrast, LMW-HA, which accumulates in response to oxidative stress, tissue injury, or enzymatic degradation, exerts pro-inflammatory and pro-fibrotic effects. Acting as a damage-associated molecular pattern (DAMP), LMW-HA engages innate immune receptors, particularly TLR2 and TLR4, leading to activation of NF-κB, cytokine production, and immune cell infiltration into liver tissue [25]. Beyond its interaction with TLRs, LMW-HA also binds to CD44 and RHAMM, amplifying inflammatory and remodeling processes. This engagement promotes inducible nitric oxide synthase (iNOS) expression, enhances T cell activation and proliferation, induces adhesion molecule upregulation, and stimulates the migration and proliferation of fibroblasts and endothelial cells, key cellular events driving ECM remodeling and fibrogenesis [26]. Therefore, the molecular weight-dependent duality of HA in receptor engagement and functional outcomes underscores the biological complexity of this naturally occurring glycosaminoglycan. Moreover, it highlights HA’s context-dependent role in shifting from a homeostatic mediator under healthy conditions to a pathogenic driver in chronic liver disease.

## 3. HA in the Hepatic Sinusoidal Niche

Understanding the role of HA in the liver requires consideration of the unique microarchitecture and composition of the hepatic ECM, which, unlike the dense interstitial ECM found in most organs, is relatively sparse, highly dynamic, and spatially compartmentalized [27]. The unique organization of hepatic architecture enables continuous and precisely regulated interactions among ECM components and diverse cell populations, supporting metabolic, immunological, and structural homeostasis. A key anatomical niche for these interactions is the space of Disse, a perisinusoidal compartment separating hepatocytes from the fenestrated LSECs. This space contains extracellular matrix molecules, including HA, collagen types IV and VI, fibronectin, laminins, and proteoglycans, as well as pro-fibrogenic HSCs and resident macrophages (KCs) [28]. Within this framework, as commented above, HA not only contributes to hydration and viscoelasticity, but also plays an essential regulatory role by interacting with cell surface receptors, such as CD44 and RHAMM, thereby modulating cell adhesion, migration, signaling, and responses to mechanical cues [29]. Specifically, HA is particularly enriched in the space of Disse, where it works as a dynamic interface between hepatocytes and LSECs, modulating multiple cell-specific functions. Among them, HA regulates the activation and migration of HSCs, supports KC adhesion and polarization, and affects the phenotype and immune responses of liver-resident and infiltrating macrophages via CD44-mediated signaling. Additionally, HA contributes to sinusoidal immune surveillance by interacting with immune cells such as T lymphocytes and dendritic cells (DCs), modulating their trafficking and activation states [11,30]. Particular attention should be paid to the unique phenotype and function of LSECs, which receive blood from both the portal vein and hepatic artery and serve as key regulators of hepatic vascular homeostasis. The pericellular matrix of LSECs, including a specialized HA-rich glycocalyx, is notably more complex and functionally versatile than endothelial cells in other anatomical locations of the body. This HA-enriched layer is critical in controlling sinusoidal permeability, mechanotransduction signaling in response to shear stress, and immune regulation. It also plays a direct role in antigen presentation and in mediating leukocyte rolling and adhesion via interaction with CD44 and other HA-binding receptors [31].

## 4. HA Metabolism and Clearance in the Liver

HA is a linear, non-sulfated glycosaminoglycan composed of repeating disaccharide units of *N*-acetyl-D-glucosamine (GlcNAc) and D-glucuronic acid (GlcUA) linked by alternating β-1,3 and β-1,4 glycosidic bonds. HA can reach exceptionally high molecular weights, up to 10^8^ Da, allowing it to form highly hydrated, viscoelastic structures within the ECM [32]. Unlike other glycosaminoglycans, HA is not synthesized in the Golgi apparatus nor attached to a core protein; instead, it is directly extruded into the extracellular space by membrane-bound hyaluronan synthases. Degradation is controlled enzymatically or by non-enzymatic mechanisms associated with oxidative damage. Importantly, sinusoidal endothelial cells of the lymph node, spleen, and the liver are the main sites for HA clearance from the circulation and are responsible for the rapid turnover, within 2–5 min, observed in the bloodstream [33,34].

### 4.1. HA Synthesis: Isoform-Specific Roles

HA is synthesized at the inner surface of the plasma membrane from cytoplasmic nucleotide sugars, uridine diphosphate (UDP)-GlcUA, and UDP-GlcNAc, by three membrane-bound hyaluronan synthase (HAS) isoforms: HAS1, HAS2, and HAS3. These isoenzymes differ in their enzymatic efficiency, regulation, and the molecular weight of the HA polymers they generate [35]. Among them, HAS2 is the predominant isoform under physiological conditions and is mainly responsible for producing HMW-HA (>2 × 10^3^ kDa), which exhibits anti-inflammatory, anti-angiogenic, and homeostatic properties in multiple tissues, including the liver [36]. Importantly, HAS2 is highly inducible by metabolic stressors, hypoxia, and transforming growth factor beta (TGF-β) signaling, key cues in hepatic regeneration and fibrogenesis [37]. From an evolutionary perspective, a recent study by Zhang et al. in the non-aging naked mole-rat model further underscored the physiological importance of HAS2 and HMW-HA by linking their sustained tissue expression to exceptional longevity in vertebrates, suggesting a conserved role in maintaining tissue integrity and resilience across species [38].

HAS1 also produces HMW-HA but displays low basal activity. Its enzymatic function requires relatively high concentrations of UDP-GlcNAc and UDP-GlcUA, making it particularly responsive to glycemic stress or altered nucleotide sugar availability [39]. Under such conditions, HAS1 contributes to the assembly of a dense pericellular HA coat, often associated with CD44 engagement, which regulates cell proliferation, adhesion, and immune cell recruitment [40].

In contrast, HAS3 primarily synthesizes HA polymers of lower molecular weight than those generated by HAS2, ranging approximately from 20 kDa to 2000 kDa [41]. Although HAS3 displays low basal activity under homeostatic conditions, its expression is inducible under pro-inflammatory stimuli. Notably, interleukin-1 beta (IL-1β) has been shown to upregulate HAS3 transcription, resulting in increased production of LMW-HA fragments that stimulate chemokine and cytokine release, thereby amplifying inflammatory responses [42]. While direct evidence in hepatic tissue is currently limited, the inducible nature of HAS3 under inflammatory conditions suggests that it may contribute to liver inflammation and fibrosis. Further liver-specific studies are needed to clarify its role in chronic liver disease progression.

During the progression of liver fibrosis, HA accumulation is primarily driven by the activation of hepatic HSCs, which upregulate HAS, particularly HAS2, in response to profibrogenic cues such as TGF-β, oxidative stress, and hypoxia. Yang et al. [37] demonstrated that both HAS2 and HA are markedly overexpressed in fibrotic human livers and in multiple murine models of liver fibrosis. Using HSC-specific genetic manipulation, these authors showed that deletion of HAS2 significantly reduced HA production and fibrogenesis, while HAS2 overexpression led to increased HA deposition, HSC activation, and exacerbated fibrosis. Mechanistically, this profibrotic effect was shown to be mediated by a regulatory axis involving microRNA-200c (miR-200c), which negatively controls HAS2 expression at the post-transcriptional level. During chronic liver injury, miR-200c levels were significantly downregulated, relieving its repression of HAS2 and thereby enabling pathological HA accumulation [43].

Interestingly, HAS expression shows cell- and tissue-specific regulation. In visceral adipose tissue (VAT) from individuals with obesity, HAS1 is selectively upregulated, while HAS2 and HAS3 remain largely unchanged, suggesting that HAS1-driven HA synthesis in adipose tissue may contribute to systemic inflammation and the development of MASLD [25].

### 4.2. HA Degradation and Clearance

HA is degraded through both enzymatic and non-enzymatic pathways. Among the six described human hyaluronidase genes, HYAL1 and HYAL2 play the most prominent roles in HA catabolism. HYAL2, anchored to the plasma membrane via a glycosyl-phosphatidylinositol (GPI) linkage, initiates degradation by cleaving HMW-HA into intermediate-sized fragments. These fragments are then internalized and further hydrolyzed by HYAL1, a soluble lysosomal enzyme that completes the catabolic process within endolysosomal compartments [44].

This sequential degradation is crucial for maintaining HA homeostasis and preventing extracellular accumulation. In the liver, LSECs play a central role in systemic HA clearance through high-affinity, receptor-mediated endocytosis via stabilin-2, also known as HARE (hyaluronan receptor for endocytosis) [17,45]. Under physiological conditions, this pathway ensures tight regulation of plasma HA levels. However, during chronic liver disease, LSEC function becomes progressively impaired. Capillarization and downregulation of stabilin-2 reduce HA uptake, contributing to elevated serum HA concentrations that correlate with fibrotic burden and disease progression [31].

In addition to enzymatic degradation, HA is highly susceptible to non-enzymatic fragmentation, particularly under oxidative stress conditions, a hallmark of chronic liver injury [46,47]. Reactive oxygen species (ROS) can indiscriminately cleave HA chains, generating biologically active LMW HA fragments with pro-inflammatory effects engaging, as previously mentioned, TLR2 and TLR4 on liver cells [48].

Overall, a tightly regulated balance between HA synthesis, degradation, and clearance is essential for maintaining hepatic homeostasis. In chronic liver disease, this equilibrium is disrupted, leading to HA accumulation and compositional shifts in HA pools that promote immune dysregulation, fibrogenesis, and progression toward fibrosis, cirrhosis and HCC. Additionally, recent findings reveal that HA is not confined to the extracellular space. Intracellular pools of HA have been detected in the cytoplasm and nucleus, where they may contribute to processes such as cell cycle regulation, RNA splicing, and autophagy [49]. These insights expand our understanding of HA biology, suggesting it may also modulate intracellular mechanisms relevant to liver pathology.

## 5. HA as an Immune Modulator in Liver Fibrosis

Inflammation is a central driver of hepatic fibrogenesis in chronic liver injury. Persistent inflammatory signals promote HSC trans-differentiation into myofibroblast-like cells that secrete excessive extracellular matrix components, leading to architectural distortion and loss of organ function [50,51]. Although the liver has an extraordinary regenerative capacity and maintains immunological tolerance under physiological conditions, these functionalities progressively deteriorate as fibrosis advances [52]. Once cirrhosis and its complications become clinically apparent, reversal of liver injury is rarely achievable, seriously compromising patient prognosis [53]. As previously discussed, HA has emerged as a key immunomodulatory molecule within the fibrotic microenvironment, influencing both innate and adaptive immune responses. In early stages of liver injury, HA may contribute to tissue repair; however, under sustained injury and chronic inflammation, HA molecules can promote immune cell activation, amplifying inflammatory circuits and supporting tumor-promoting remodeling [4].

### 5.1. Innate Immune Response

**Figure 1:**
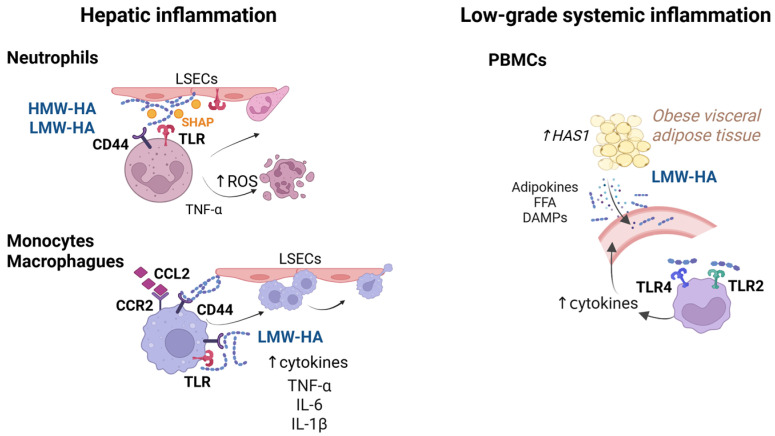
Proinflammatory role of hyaluronic acid (HA) in innate immune activation. (**Left panel**): In the hepatic sinusoid, HA activates neutrophils and monocyte-derived macrophages through CD44 and Toll-like receptors (TLRs), promoting cytokine release, endothelial adhesion, and migration across the sinusoidal barrier. It also amplifies reactive oxygen species (ROS) generation by inducing apoptotic cell death of neutrophils primed by tumor necrosis factor alpha (TNF-α). (**Right panel**): Systemically, low-molecular-weight HA (LMW-HA) activates peripheral blood mononuclear cells (PBMCs) via TLRs in the context of obesity-associated inflammation. Abbreviations: CCL2, C-C motif chemokine ligand 2; CCR2, C-C chemokine receptor type 2; CD44, lymphocyte homing receptor; DAMPs, damage-associated molecular patterns; FFAs, free fatty acids; HA, hyaluronic acid; HAS1, hyaluronan synthase 1; HMW-HA, high-molecular-weight hyaluronic acid; IL-1β, interleukin-1 beta; IL-6, interleukin-6; LMW-HA, low-molecular-weight hyaluronic acid; LSECs, liver sinusoidal endothelial cells; PBMCs, peripheral blood mononuclear cells; ROS, reactive oxygen species; SHAP, serum-derived hyaluronan-associated protein; TLR2, Toll-like receptor 2; TLR4, Toll-like receptor 4; TNF-α, tumor necrosis factor alpha. Created in BioRender. JCE Lab. (2025) https://BioRender.com/zxrl35j (accessed on 12 October 2025).

HA plays a pivotal role in orchestrating the early immune response to liver injury by facilitating the recruitment of leukocytes to sites of tissue damage (Figure 1). Among the first responders are neutrophils, which migrate into the liver through the specialized microvasculature of the hepatic sinusoids. McDonald and colleagues [51,52] demonstrated that during endotoxin-derived liver inflammation in mice, HA accumulates on the luminal surface of LSECs, promoting neutrophil adhesion via CD44 receptors expressed on neutrophils but not on the endothelium. Further studies from this group showed that endothelial TLR4 activation enhances neutrophil adhesion by inducing serum-derived hyaluronan-associated protein (SHAP), which increases the binding affinity of HA for neutrophil CD44 [54,55]. This SHAP-dependent mechanism represents a divergence from the classical selectin/integrin-mediated rolling observed in systemic vasculature, highlighting the immunologically unique microenvironment of liver sinusoids. The nature of the hepatic insult, whether infectious, driven by pathogen-associated molecular patterns (PAMPs), or sterile, driven by DAMPs as in MASLD, critically influences the recruitment, activation, and functional polarization of neutrophils and other innate immune cells. These distinctions shape not only the inflammatory milieu but also the immunomodulatory role of HA. In this context, HA molecules may differentially regulate leukocyte dynamics depending on the underlying etiology and immune status of the liver microenvironment [56].

Beyond its role in leukocyte recruitment, both LMW-HA and HMW-HA fragments actively modulate neutrophil activation and function. Niemietz and colleagues [57] demonstrated in ex vivo experiments using peripheral blood cells from healthy donors, that HA, irrespective of its molecular mass, primes neutrophils to produce ROS through the activation of the p38 MAPK signaling pathway. A subsequent ex vivo study by the same group using polymorphonuclear cells from healthy individuals showed that HMW-HA, particularly in the presence of tumor necrosis factor-alpha (TNF-α) priming, enhances ROS production in neutrophils and accelerates apoptotic cell death [58]. In the liver, where elevated levels of HA and TNF-α frequently coexist in chronic inflammatory states such as MASLD and viral hepatitis, this synergistic mechanism may intensify neutrophil-mediated cytotoxicity, thereby contributing to hepatocellular injury and the progression of fibrosis [59].

**Figure 2:**
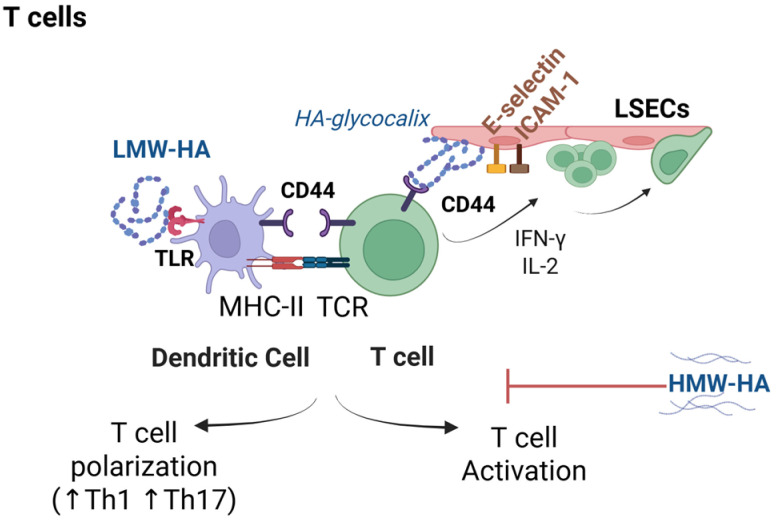
Role of hyaluronic acid (HA) in the modulation of adaptive immune responses. HA expressed on the sinusoidal endothelium facilitates the adhesion and transendothelial migration of activated T lymphocytes through interactions between endothelial HA and the CD44 receptor on T cells. In addition, CD44 colocalizes within lipid rafts containing the T cell receptor (TCR)–CD3 complex at the immunological synapse between dendritic cells and T cells, thereby stabilizing intracellular signaling and promoting T cell activation and proliferation. Moreover, while low-molecular-weight HA (LMW-HA) fragments promote T helper 1 (Th1) and Th17 polarization, high-molecular-weight HA (HMW-HA) fragments attenuate these responses in vitro and redirect signaling toward immunological tolerance. Abbreviations: CD3, cluster of differentiation 3; CD44, lymphocyte homing receptor; E-selectin, endothelial selectin; HA, hyaluronic acid; HMW-HA, high-molecular-weight hyaluronic acid; ICAM-1, intercellular adhesion molecule-1; IFN-γ, interferon gamma; IL-2, interleukin-2; LMW-HA, low-molecular-weight hyaluronic acid; LSECs, liver sinusoidal endothelial cells; MHC II, major histocompatibility complex class II; Th1, T helper type 1 cell; Th17, T helper type 17 cell; TCR, T cell receptor; TLR, Toll-like receptor. Created in BioRender. JCE Lab (2025) https://BioRender.com/4yyce61 (accessed on 12 October 2025).

In vivo studies in experimental models of non-alcoholic steatohepatitis assessing CD44 deficiency suggest that HA molecules exert modulatory actions on hepatic macrophages, including resident KCs and infiltrating monocyte-derived macrophages (Figure 2). In particular, CD44-deficient mice show reduced macrophage responsiveness to inflammatory stimuli and a skewing toward a more anti-inflammatory phenotype, thereby attenuating liver inflammation and fibrosis [30]. Complementary in vitro studies indicate that the molecular size of HA is a key determinant of macrophage polarization. Specifically, HMW-HA (>1250 kDa) has been shown to induce M2-like polarization of unstimulated human macrophages, as evidenced by increased expression of CD163, CD206, and IL-10, whereas smaller HA fragments lacked this effect [7]. Moreover, studies in other experimental systems support a broader role of HA–receptor interactions in macrophage polarization: the expression of CD44 varies with macrophage activation state [60] and HA/RHAMM signaling can drive macrophages toward pro-inflammatory phenotypes in vitro [61].

In addition to modulating macrophage polarization, a preclinical study by Egan and colleagues demonstrated that HA binding to CD44 promotes integrin activation and stabilizes monocyte adhesion to LSECs, facilitating monocyte transendothelial migration. This process is further coordinated by chemokine signaling, particularly by the CCL2-CCR2 axis, which synergizes with HA-CD44 interactions to guide monocyte trafficking and retention within inflamed hepatic tissue [62].

Beyond cell trafficking, HA-CD44 signaling has also been shown to shape the immunological function of hepatic myeloid populations. In both murine and human systems, CD44 engagement on activated HSC promotes the induction of myeloid-derived suppressor cells (MDSCs), fostering an immunosuppressive microenvironment. This mechanism supports immune surveillance during early stages of liver disease but may also contribute to immune evasion and tumor progression in advanced hepatic malignancies [63,64].

Fragmented LMW-HA, which accumulates in liver injury and chronic inflammation, also acts as a DAMP by activating TLR2 and TLR4 on macrophages, amplifying cytokine release (TNF-α, IL-6, and IL-1β) and contributing to a self-perpetuating inflammatory loop [11,48]. In contrast, HMW-HA exerts immunomodulatory effects that favor the resolution of inflammation. HMW-HA (intermediate size from 500 to 1000 kDa) has been shown to promote an M2-like macrophage phenotype and to support tissue repair, partly by suppressing TLR-mediated activation [65]. Consistently, Maharjan et al. demonstrated that HMW-HA suppresses the differentiation of human fibrocytes, pro-fibrotic and inflammatory monocyte-derived cells, while LMW-HA promotes their maturation [66]. These findings underscore the context-dependent, size-specific immunological effects of HA, suggesting that HA molecular weight is a key determinant in modulating the inflammatory landscape of the liver.

### 5.2. Adaptive Immune Response

HA contributes to adaptive immunity from the earliest stages of T cell recruitment (Figure 2). A seminal study in mice published by deGrendele et al. demonstrated that HA expressed on the vascular endothelium facilitates CD44-dependent adhesion and transendothelial migration of activated T cells [67]. Subsequent studies have corroborated these findings in various models of chronic inflammation, including liver disease, where HA-CD44 interactions, either alone or in cooperation with adhesion molecules such as E-selectin and intracellular adhesion molecule-1 (ICAM-1), facilitate the infiltration and retention of effector T cells within inflamed tissues [18,68,69] (Figure 2).

Beyond its role on T cell recruitment, HA-CD44 signaling plays a pivotal role in T cell activation and functional differentiation. Upon antigen recognition, CD44 is recruited to the immunological synapse, co-localizing with lipid rafts and the TCR-CD3 complex, thereby stabilizing interactions between DCs and T cells. This spatial organization facilitates efficient T cell receptor signaling and sustained contact with antigen-presenting cells (Figure 2). Notably, in a preclinical study by Hegde and colleagues, CD44-deficient DCs exhibit impaired ability to stimulate T cell proliferation and cytokine production, such as IL-2 and interferon-gamma (IFN-γ) [70]. Mechanistically, CD44 interacts with the ezrin–radixin–moesin family of proteins, anchoring it to the actin cytoskeleton and promoting cytoskeletal remodeling necessary for synapse formation and T cell polarization [71].

The molecular weight of HA also critically influences its immunomodulatory effects on the adaptive immune system. Preclinical studies have indicated that LMW-HA promotes Th1 and Th17 polarization by inducing major histocompatibility complex class II (MHC II) and co-stimulatory molecule expression (CD80 and CD86) on DCs via TLR4-dependent signaling [14,72]. In contrast, HMW-HA has been shown to suppress T cell proliferation and cytokine production in vitro, suggesting a potential role in promoting immune tolerance [3,73].

In the context of chronic liver inflammation, these size-dependent effects of HA are particularly relevant. Early evidence from a T-cell-mediated liver injury model induced by concanavalin A demonstrated that administration of HMW-HA (900 kDa) significantly reduced plasma levels of pro-inflammatory cytokines, including TNF-α and IFN-γ. In contrast, LMW-HA (250 kDa) failed to confer similar protective effects [74].

## 6. HA in Chronic Liver Pathology: From MASLD to HCC

MASLD, a term recently redefined to reflect its metabolic roots, is now recognized as the most prevalent chronic liver disease worldwide, affecting approximately one-third of adults. Individuals at increased risk are identified by the presence of at least one of the following criteria: type 2 diabetes mellitus (T2DM), obesity, or persistently elevated aminotransferase levels in the absence of secondary causes of steatosis [75].

The pathogenesis of MASLD involves a complex interplay between hepatic and systemic metabolic stressors. Among extrahepatic contributors, dysfunctional VAT plays a pivotal role. It becomes hypertrophic, insulin-resistant, and inflamed, leading to the release of pro-inflammatory adipokines (e.g., TNF-α and IL-6), saturated fatty acids, and DAMPs such as high-mobility group box 1 (HMGB1), extracellular adenosine triphosphate (ATP), and HA. This constellation of circulating factors contributes to a systemic, chronic low-grade inflammatory state that exacerbates hepatic lipotoxicity, oxidative stress, and immune activation, thereby accelerating fibrogenic remodeling [59,76].

Emerging evidence highlights the regulatory crosstalk between these metabolic stressors and HA synthesis pathways. For instance, extracellular ATP has been shown to upregulate HA production by activating HAS2 via purinergic P2Y_2_ receptor-mediated Ca^2+^ signaling and downstream MAPK activation in a human cell line of keratinocytes [77]. In parallel, HMGB1 contributes to hepatic fibrogenesis by activating pattern recognition receptors, such as TLR4 and receptor for advanced glycation endproducts (RAGE), on HSCs, which are the major sources of HA in a fibrotic liver. Although the direct effects of HMGB1 on HA metabolism remain to be fully elucidated, it likely modulates HA indirectly by promoting an inflammatory microenvironment [78]. Moreover, HA homeostasis may be influenced by the systemic redox balance. Uric acid, a DAMP released during tissue stress, can scavenge ROS, protecting HA chains from oxidative fragmentation and potentially altering ECM architecture during chronic liver injury [79]. Collectively, these molecular interactions position HA as a mediator that links systemic metabolic dysregulation with local hepatic inflammation and fibrogenesis.

HA emerges not only as a biomarker of fibrosis severity, with serum levels and hepatic deposition correlating with disease stage across the MASLD spectrum [80,81], but also as a bioactive effector molecule in sterile metabolic dysfunction. Notably, increasing evidence supports the notion that HA metabolism is systemically dysregulated in human metabolic disease. Romo et al. reported increased VAT expression of HAS1 and elevated circulating levels of LMW-HA in individuals with obesity and metabolic dysfunction, suggesting that adipose tissue may contribute to a pro-inflammatory HA pool in the bloodstream [25]. In parallel, Kang et al. demonstrated that excessive HA accumulation in a high-fat diet mouse model aggravated insulin resistance and metabolic dysregulation, reinforcing a mechanistic link between HA synthesis and metabolic impairment [82]. These findings point to a role for HA as a systemic amplifier of chronic inflammation, upstream of liver injury. In support of this, LMW-HA fragments have been shown to activate TLR2 and TLR4 signaling on monocytes and macrophages and peripheral blood mononuclear cells, thereby linking adipose tissue inflammation to hepatic immune activation (Figure 2) [11,25].

Together, these observations position HA as a systemic amplifier of low-grade inflammation in metabolic disease, while underscoring the importance of HA size and receptor context when interpreting its effects across the MASLD-HCC continuum.

**Figure 3:**
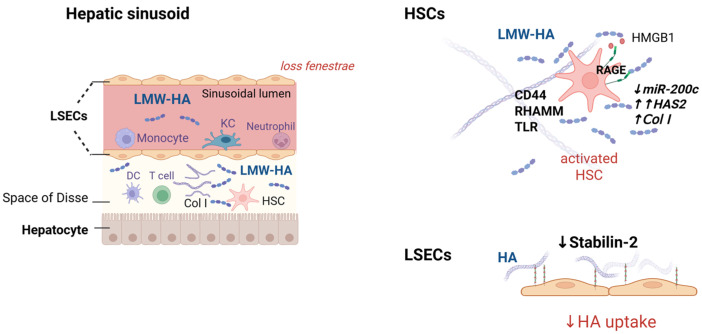
Role of hyaluronic acid (HA) in liver fibrosis. (**Left panel**): Representation of a hepatic sinusoid under chronic injury showing hepatocytes; the space of Disse enriched in extracellular matrix components (collagens and HA); activated hepatic stellate cells (HSCs); infiltrating leukocytes; tissue macrophages (Kupffer cells); and liver sinusoidal endothelial cells (LSECs). Low-molecular-weight HA (LMW-HA) fragments circulate in the sinusoidal lumen together with inflammatory leukocytes. (**Right panel**): In activated HSCs, downregulation of microRNA-200c (miR-200c), a post-transcriptional repressor of hyaluronan synthase 2 (HAS2), leads to enhanced HA synthesis and accumulation. Along with the upregulation of HA-binding receptors such as RHAMM and the pro-inflammatory receptor for advanced glycation end products (RAGE), this promotes HA-driven DAMP signaling that sustains HSC activation and collagen deposition. In parallel, LSECs, the principal site of systemic HA clearance through stabilin-2-mediated endocytosis, become dysfunctional during chronic liver disease: they undergo capillarization, lose fenestrae, and show reduced stabilin-2 expression, thereby impairing HA uptake and contributing to elevated circulating HA levels. Abbreviations: CD44, lymphocyte homing receptor; Col I, collagen type I; DAMPs, damage-associated molecular patterns; DCs, dendritic cells; HA, hyaluronic acid; HAS2, hyaluronan synthase 2; HMGB1, high-mobility group box 1; HSCs, hepatic stellate cells; KC, Kupffer cell; LMW-HA, low-molecular-weight hyaluronic acid; LSECs, liver sinusoidal endothelial cells; miR-200c, microRNA-200c; RAGE, receptor for advanced glycation end products; RHAMM, receptor for hyaluronan-mediated motility; TLR, Toll-like receptor. Created in BioRender. JCE Lab (2025) https://BioRender.com/htg4idt (accessed on 12 October 2025).

As previously described, HSCs are the major source of extracellular matrix in chronic liver injury, and their transdifferentiation into myofibroblast-like cells represents a key pathogenic event underlying fibrosis progression in MASLD and other chronic liver diseases. Under physiological conditions, HSCs remain quiescent and contribute to ECM turnover. However, in response to metabolic stressors, including lipotoxicity, hyperglycemia, oxidative damage, and inflammatory signals, they transdifferentiate into myofibroblast-like cells that proliferate and secrete fibrillar collagens, fibronectin, and HA, thereby driving fibrosis and architectural disruption [83]. As discussed earlier, the HA-producing enzyme HAS2 is particularly upregulated in activated HSCs and plays a central role in driving fibrotic remodeling through the HA-CD44-neurogenic locus notch homolog protein 1 (NOTCH1) axis and miR-200c (Figure 3) [37,43].

As further discussed in the following paragraphs, in addition to HSCs, other non-parenchymal hepatic cells, such as LSECs and hepatic progenitor cells (HPCs), may also engage with the HA axis and contribute to ECM remodeling during MASLD progression.

Endothelial dysfunction is also a hallmark of MASLD, contributing to hepatic fibrogenesis and, in advanced stages, to vascular complications within the hepatic tissue. In the liver, chronic metabolic stress induces capillarization of LSECs, characterized by the loss of fenestrae, formation of a continuous basement membrane, and adoption of a pro-inflammatory and pro-thrombotic phenotype. These changes are accompanied by aberrant accumulation of HA in the space of Disse, which impairs hepatocyte-endothelial communication and facilitates leukocyte adhesion and transmigration (Figure 3) [54,84].

Disruption of the endothelial glycocalyx, particularly the loss of HMW-HA, which predominates under physiological conditions, impairs mechanosensitive signaling, reduces endothelial nitric oxide synthase (eNOS) activation, and promotes endothelial dysfunction. This leads to sinusoidal vasoconstriction, reduced perfusion, and local hypoxia, which further activate inflammatory and fibrotic pathways [85]. As intrahepatic vascular resistance rises, sinusoidal dysfunction translates into portal hypertension and, at advanced stages, ascites, reflecting the hemodynamic consequences of endothelial injury [86]. Experimental studies have shown that enzymatic removal of HA decreases flow-induced eNOS activation in endothelial cells, both in vitro and in vivo, supporting its role in shear-stress-dependent nitric oxide (NO) production [87,88]. Although these studies implicate HA in modulating eNOS activity, direct mechanistic evidence in hepatic endothelium remains limited, and some of the observed effects may be secondary to broader alterations in oxidative stress and inflammatory tone.

The hypoxic and proinflammatory microenvironment induced by LSEC dysfunction promotes HSC activation, driving angiogenic and fibrogenic cascades, notably through vascular endothelial growth factor (VEGF) and platelet-derived growth factor (PDGF) signaling pathways [89]. Interestingly, experimental evidence suggests that HA can promote angiogenesis independently of VEGF. In extrahepatic models, high concentrations of HA stimulate endothelial migration and tube formation through RHAMM- and transforming growth factor beta receptor I (TGF-βRI)-dependent pathways, upregulating plasminogen activator inhibitor-1 (PAI-1), a key regulator of ECM remodeling and neovascularization [90]. While such mechanisms are biologically plausible in the liver, their relevance in the context of MASLD remains to be formally demonstrated in vivo.

Another emerging component of the stromal microenvironment is the hepatic progenitor cell (HPC) niche. In chronic liver injury, HPC activation and CD44 expression are frequently observed in periportal regions and near HA-enriched fibrotic septa, suggesting a potential spatial and functional interaction [91]. Although direct mechanistic evidence in the liver is still limited, studies from other organ systems have demonstrated that HA regulates progenitor cell behavior through engagement of CD44 and RHAMM, influencing cell proliferation, migration, and differentiation [92]. These findings raise the possibility that HA may influence HPC fate decisions during fibrotic remodeling in MASLD, although this hypothesis requires further experimental validation in hepatic models.

Overall, beyond serving as a structural component or biomarker, HA emerges as a dynamic modulator of stromal cell behavior, influencing endothelial integrity, hepatic stellate cell activation, and progenitor cell fate. This cumulative disruption of hepatic architecture and immune balance not only exacerbates fibrosis but also lays the groundwork for malignant transformation.

### HA-Mediated Immune Reprogramming in HCC Progression

**Figure 4:**
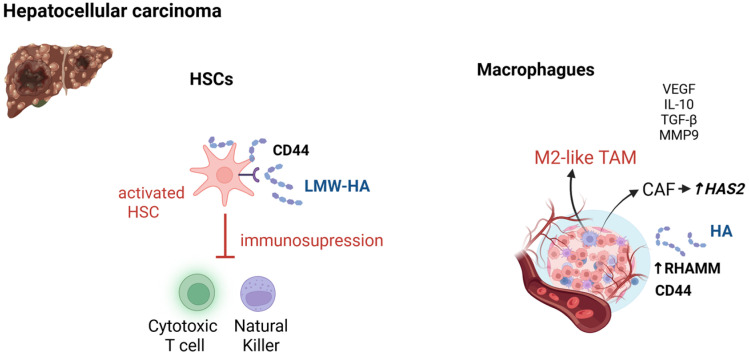
Role of hyaluronan (HA) in hepatic carcinogenesis. (**Left panel**): In the peritumoral niche, activated hepatic stellate cells (HSCs) contribute to LMW-HA accumulation and signaling through CD44, RHAMM, and TLRs. CD44-HA interactions promote the expansion and activation of cytotoxic T lymphocytes and natural killer (NK) cells. (**Right panel**): The tumor-associated macrophage (TAM) compartment plays a central role in the tumorigenesis process. Accumulated LMW-HA supports TAM maintenance and polarization toward an immunosuppressive phenotype, driving the secretion of cytokines and mediators that sustain immune evasion, angiogenesis, and ECM remodeling. In this tumor milieu, RHAMM expression is upregulated, and cancer-associated fibroblasts (CAFs) further amplify this loop by up-regulating hyaluronan synthase 2 (HAS2), enhancing HA synthesis and promoting tumor progression. Abbreviations: CAFs, cancer-associated fibroblasts; CD44, lymphocyte homing receptor; ECM, extracellular matrix; HA, hyaluronic acid; HAS2, hyaluronan synthase 2; HCC, hepatocellular carcinoma; HSCs, hepatic stellate cells; IL-10, interleukin-10; LMW-HA, low-molecular-weight hyaluronic acid; MMP-9, matrix metalloproteinase-9; NK, natural killer; RHAMM, receptor for hyaluronan-mediated motility; TAMs, tumor-associated macrophages; TGF-β, transforming growth factor beta; TLR, Toll-like receptor; VEGF, vascular endothelial growth factor. Created in BioRender. JCE Lab (2025) https://BioRender.com/qkgxpzm (accessed on 12 October 2025).

Compared with adjacent non-tumor liver tissue, clinical studies have shown that HCC commonly exhibits peritumoral or stromal HA enrichment together with increased expression of HA receptors, particularly CD44 and RHAMM, features linked to invasion, immune suppression, and poorer outcomes (Figure 4) [13,93]. HA actively contributes to the immunological and stromal remodeling that characterizes HCC development, particularly in the context of metabolic liver disease. Through engagement with CD44 and other receptors, HA regulates tumor cell motility, epithelial-to-mesenchymal transition, and resistance to apoptosis and chemotherapy, fostering malignant progression. In both human and murine models of HCC, CD44 signaling on activated HSCs promotes the expansion of MDSCs, which inhibit cytotoxic T lymphocyte (CTL) and natural killer (NK) cell responses, thus reinforcing an immune-suppressive niche [63,64].

In malignant hepatocytes, CD44 overexpression correlates with poor clinical outcomes and facilitates tumor progression by sustaining receptor tyrosine kinase signaling, repressing pro-apoptotic Fas expression, and maintaining cancer stem cell features through crosstalk with epidermal growth factor receptor (EGFR), mesenchymal–epithelial transition factor (c-Met), platelet-derived growth factor receptor beta (PDGFR-β), and TGF-βRI pathways [12,94,95]. In parallel, increasing attention has been given to RHAMM, another HA receptor implicated in HCC progression. Immunohistochemical analyses have shown that RHAMM is upregulated in tumor–node regions of HCC specimens and that high RHAMM expression correlates with reduced disease-free and overall survival following surgical resection, suggesting it may serve as an independent prognostic marker [13]. Notably, Wu and colleagues showed in a murine HCC model that *HMMR* knockout suppresses tumor growth and enhances macrophage-mediated phagocytosis; mechanistically, RHAMM assembles a cytoplasmic complex that activates (focal adhesion kinase) FAK-SRC-NF-κB signaling to sustain CD47-mediated anti-phagocytic signaling independently of CD44. Genetic *HMMR* loss also augmented anti-PD-1 efficacy, and RHAMM high/CD47 high human tumors were associated with poorer outcomes [96]. These findings support the emerging role of RHAMM in modulating tumor invasiveness [97] and underscore its potential as a therapeutic target in HA-rich hepatic tumors.

Thus, whereas HA accumulation in chronic liver disease primarily reflects extracellular matrix remodeling and impaired clearance, in HCC the HA-rich stroma and heightened HA–receptor signaling create permissive niches that foster epithelial–mesenchymal transition, immune evasion, and therapy resistance. However, further studies are needed to elucidate the size-dependent effects of HA fragments within the tumor microenvironment and their specific contribution to hepatocarcinogenesis [98].

A pivotal axis in HA-mediated immune modulation involves tumor-associated macrophages (TAMs), which represent a dominant immune population in the HCC microenvironment and play a central role in sustaining immunosuppression and tumor progression (Figure 4) [99]. These cells typically exhibit an M2-like phenotype, characterized by the secretion of IL-10, TGF-β, VEGF, and MMP9, which collectively support immune evasion, extracellular matrix remodeling, and angiogenesis (Figure 3) [100]. Accumulation of LMW-HA during ECM remodeling contributes to this pro-tumoral polarization. In vitro and preclinical studies have shown that size-specific HA fragments can engage TLR4 on human monocytes, leading to decreased miR-935 and increased C/EBPβ expression, which in turn promote an M2-like transcriptional profile characterized by the upregulation of IL-10 and ARG1 [65]. Notably, this signaling differs from canonical TLR4 activation by lipopolysaccharide (LPS), as intermediate-sized, polydisperse HA preparations (ranging approximately from 50 to 1000 kDa), but not LPS, induced M2-like macrophage differentiation, highlighting the context-dependent immunomodulatory role of HA [65]. Furthermore, an HA-rich stroma has been shown to facilitate macrophage infiltration and neovascularization, as evidenced by murine models in which exogenous HA enhanced TAM recruitment and vascular remodeling [101] Although direct evidence in HCC is limited, experimental models in glioblastoma and other solid tumors have demonstrated that disruption of HA synthesis or blockade of HA-CD44 interactions reprograms TAMs toward a pro-inflammatory M1 phenotype, thereby restoring antitumor immunity and reducing tumor growth [102]. These findings suggest that HA-CD44 signaling contributes to the maintenance of TAM-driven immunotolerance in HCC, and that targeting this axis may offer therapeutic benefit.

Finally, in the context of MASLD-associated fibrosis, upregulation of HAS2 in activated HSCs leads to excessive HA deposition within the fibrotic stroma. This accumulation fosters an immunosuppressive and pro-tumorigenic hepatic microenvironment by modulating extracellular matrix composition and immune cell infiltration (Figure 4). In vivo studies have shown that genetic or pharmacologic inhibition of HAS2 reduces stromal HA levels, alters the immune landscape, and significantly attenuates HCC growth, underscoring the HAS2-HA axis as a promising therapeutic target in liver cancer [103]. Although HCC can arise without cirrhosis in MASLD [104], current data do not demonstrate consistent differences in HA fragment-size distributions or receptor expression profiles compared with cirrhotic HCC, and further systematic, etiology-stratified studies are needed.

In summary, the convergence of fibrogenic, immunomodulatory, and angiogenic mechanisms in chronic liver disease positions HA as a critical driver of malignant progression in MASLD-associated HCC, making it a rational target for therapeutic disruption.

## 7. Therapeutic Targeting of HA Signaling and Metabolism in Liver Disease

Growing recognition of the pathogenic role of HA, particularly its LMW forms, in advanced MASLD and HCC has prompted the development of targeted strategies to mitigate its deleterious effects. These interventions aim to reduce HA accumulation, disrupt pathological HA–receptor signaling, and improve therapeutic efficacy in fibrotic and tumor-permissive hepatic microenvironments. Current approaches can be grouped into three main categories, (1) inhibition of HA synthesis, (2) enzymatic degradation of HA, and (3) blockade of HA–receptor interactions, with additional exploratory combinations with immunotherapy currently under investigation.

### 7.1. Inhibition of HA Synthesis

HA biosynthesis is driven by HAS, with HAS2 being the predominant isoform upregulated in activated HSCs during fibrogenesis. Genetic suppression of HAS2, through small interfering RNA (siRNA) or miR-200c modulation, has been shown to attenuate liver fibrosis and HA deposition in murine models [43,105].

The most extensively studied pharmacological inhibitor is 4-methylumbelliferone (4-MU), which reduces the availability of UDP-glucuronic acid, a common substrate for all HAS isoforms. In rodent models of toxic and diet-induced liver injury, 4-MU administration decreased hepatic HA content, suppressed HSC activation, and improved histological fibrosis scores without major toxicity [106]. Moreover, recent transcriptomic analyses confirm that 4-MU recapitulates the molecular effects of HAS2 knockdown [107]. Antitumor effects have also been reported in experimental HCC, where 4-MU disrupted HA-rich stromal niches and reduced tumor progression [108,109]

Despite these findings, clinical translation is limited by poor bioavailability and non-specific distribution. Liver-targeted formulations and more selective HAS2 inhibitors are under preclinical development.

### 7.2. Enzymatic Degradation of HA

Depletion of excess HA by exogenous hyaluronidases, particularly recombinant human formulations such as PEGPH20, has been explored in oncology. Preclinical models have demonstrated that HA degradation reduces interstitial pressure, enhances perfusion, and improves chemotherapeutic delivery [110]. Comparable metabolic benefits have been observed in murine models of insulin resistance, where enzymatic HA depletion improved muscle glucose uptake, vascularization, and insulin sensitivity [82].

Clinically, PEGPH20 was evaluated in the phase III HALO-301 trial involving patients with HA-high metastatic pancreatic ductal adenocarcinoma. Although the combination with nab-paclitaxel and gemcitabine increased objective response rates, it failed to extend progression-free or overall survival, and the program was discontinued due to thromboembolic risk and inconsistent efficacy [111].

Unfortunately, up to date, no clinical trials have assessed hyaluronidase therapies in liver fibrosis or HCC. Major hurdles include enzyme instability, risk of systemic side effects, and the absence of targeted hepatic delivery platforms.

### 7.3. Blockade of HA Receptors (CD44 and RHAMM)

Therapeutic strategies targeting HA receptors aim to disrupt aberrant HA-driven signaling in chronic liver disease. CD44, the principal HA receptor, is widely expressed across immune and non-parenchymal hepatic cells, including macrophages, HSCs, and cholangiocytes, where it regulates adhesion, migration, stemness, and immune evasion. Preclinical inhibition of CD44 using monoclonal antibodies or gene silencing has reduced tumor cell proliferation, MDSC recruitment, and fibrotic signaling in hepatocellular carcinoma [12]. However, its broad physiological expression complicates selective therapeutic modulation.

In contrast, RHAMM expression is more restricted, often confined to proliferating or transformed cells. Its role in HA-driven motility and angiogenesis has been implicated in tumor progression, and recent preclinical studies have shown that peptide or siRNA-based RHAMM inhibition can attenuate HCC growth and vascular remodeling in a murine HepG2 xenograft model [112]. Despite these findings, clinical translation of RHAMM-targeted strategies remains to be further investigated.

Although CD44 and RHAMM are widely recognized as amplifiers of HA-driven fibrogenic and immunomodulatory signaling in liver disease, recent evidence underscores the importance of inflammatory context in shaping HA–receptor outcomes. While LMW-HA is generally considered proinflammatory, studies by Saikia et al. [16] and You et al. [113] have shown that in models of acute liver injury triggered by alcohol or LPS, exogenous administration of small HA fragments (~35 kDa) can attenuate TLR4-mediated TNF-α signaling in Kupffer cells. In these proinflammatory settings, LMW-HA appears to act as a competitive antagonist at TLR4, mitigating hepatocyte damage.

These observations reinforce the notion that HA signaling is context-dependent, modulated by molecular weight, receptor engagement, and the nature of the inflammatory trigger. This complexity must be considered when designing HA-targeted therapies for chronic liver disease.

### 7.4. Integration with Immunotherapy and Combination Strategies

Given HA’s role in shaping an immune-suppressive, fibrotic tumor microenvironment, combining HA-targeted therapies with immunotherapies is a rational strategy. Tumors arising in steatohepatitis often exhibit poor T cell infiltration and resistance to immune checkpoint blockade. Preclinical models suggest that modulating HA content, via HAS2 inhibition or ECM degradation, can improve T cell access and potentiate anti-tumor immunity [65,110].

Of particular relevance, data published by Malehmir and colleagues, using both human samples and murine models, demonstrated that HA-CD44 signaling promotes platelet recruitment and aggregation in nonalcoholic steatohepatitis (NASH), thereby fostering a tumor-initiating niche. Inhibition of CD44 disrupted this process, attenuating early tumorigenesis and offering an immuno-thrombotic axis as a novel therapeutic target [114]. Dual inhibition of CD44 and immune checkpoints hold potential for future clinical translation, especially in HA-high or immunotherapy-resistant HCC subtypes.

## 8. Concluding Remarks and Future Perspective

HA plays a central and multifaceted role in liver pathophysiology, functioning not only as a structural component of the extracellular matrix but also as an active regulator of fibrogenic, inflammatory, and oncogenic processes. The divergent effects of high- versus low-molecular-weight HA underscore the complexity of its biological actions, from maintaining tissue architecture to promoting immune suppression, neovascularization, and malignant transformation.

Advances in understanding HA metabolism and receptor-mediated signaling, particularly via HAS2, CD44, and RHAMM, have identified new therapeutic opportunities in chronic liver disease. Preclinical strategies aimed at inhibiting HA synthesis, promoting its degradation, or blocking receptor interactions have shown efficacy in mitigating fibrosis and modulating the tumor microenvironment, especially in the context of MASLD-associated hepatocellular carcinoma.

Although not addressed in detail in this review, it is worth noting that HA’s selective affinity for CD44 is also being explored in nanomedicine-based drug delivery systems to enable targeted treatment of fibrotic or neoplastic liver tissues. Multiple studies in this growing field are investigating HA-functionalized nanoparticles to enhance therapeutic specificity and reduce systemic toxicity [115].

Collectively, these findings position HA as a modifiable determinant of liver disease progression, with emerging therapeutic relevance across the MASLD progression and HCC. Future clinical translation will depend on the development of HA-targeted agents, biomarker-driven patient stratification, and integration into rational combination therapies.

## Figures and Tables

**Table 1:** HA-Interacting Receptors Involved in Liver Physiology and Disease.

Receptor	Cellular Expression	Function in Liver	Notes	Representative References
CD44	T cells, macrophages, HSCs, LSECs	Mediates HA-dependent cell adhesion, leukocyte recruitment, MDSC induction, T cell regulation	Binding affinity modulated by HA size and SHAPs	[11,12]
RHAMM	Activated HSCs, macrophages	Promotes HA-mediated cell motility, proliferation, and inflammation	Often associated with cell migration in fibrotic and neoplastic liver environments	[1,13]
LYVE-1	LSECs, lymphatic endothelial cells	Involved in HA clearance and immune cell trafficking	Essential for hepatic and systemic HA homeostasis	[2]
TLR2	Macrophages, dendritic cells, Kupffer cells	Recognizes LMW-HA as DAMP, activates NF-κB and cytokine secretion	Functions as an innate immune sensor of ECM damage	[14]
TLR4	Macrophages, dendritic cells, Kupffer cells, HSCs	Recognizes LMW-HA, amplifies pro-inflammatory cytokine production, enhances fibrosis	Key in amplifying innate immune responses during liver injury	[15,16]
HARE/Stabilin-2	LSECs, splenic and lymph node sinusoidal endothelial cells	Mediates systemic HA clearance	High-capacity scavenger for HA and other glycosaminoglycans	[17]
ICAM-1	Endothelial cells, leukocytes	Facilitates leukocyte transmigration, interacts with HA under inflammatory conditions	Binds HA during inflammation, contributes to chronic immune cell infiltration	[18]

CD44, cluster of differentiation 44; DAMP, damage-associated molecular pattern; ECM, extracellular matrix; HA, hyaluronic acid; HARE/Stabilin-2, hyaluronan receptor for endocytosis/Stabilin-2; HSCs, hepatic stellate cells; ICAM-1, intercellular adhesion molecule-1; LMW, low molecular weight; LSECs, liver sinusoidal endothelial cells; LYVE-1, lymphatic vessel endothelial hyaluronan receptor 1; MDSC, myeloid-derived suppressor cells; NF-κB, nuclear factor kappa B; RHAMM, receptor for hyaluronan-mediated motility; SHAPs, serum-derived hyaluronan-associated proteins; TLR2, Toll-like receptor 2; TLR4, Toll-like receptor 4.

## Data Availability

No new data were created or analyzed in this study. Data sharing is not applicable to this article.
